# Bionanotechnology at microplastic and nanoplastic interfaces: detection, adsorption and degradation

**DOI:** 10.1186/s40580-026-00563-z

**Published:** 2026-07-20

**Authors:** Angrui Jiang, Wei Mao, Bowen Dai, Jung Heon Lee, Xue Bai, Juewen Liu

**Affiliations:** 1 Key Laboratory of Integrated Regulation and Resource Development on Shallow Lake of Ministry of Education, College of Environment, Hohai University, Nanjing, 210098 People’s Republic of China; 2 School of Advanced Materials Science and Engineering, Sungkyunkwan University (SKKU), Suwon, 16419 Republic of Korea; 3 Department of Chemistry, Waterloo Institute for Nanotechnology, University of Waterloo, Waterloo, ON N2L 3G1 Canada; 4 Yangtze Institute for Conservation and Development, Hohai University, Nanjing, 210098 People’s Republic of China

**Keywords:** Microplastics, Bionanotechnology, Recognition, Adsorption, Catalytic degradation

## Abstract

Micro-nano plastics (M/NPs) are pervasive environmental pollutants whose small size, persistence, and evolving surface states complicate reliable detection and remediation. As interface-dominated contaminants, their environmental behavior is largely governed by interactions at plastic–bio–nano interfaces. In this article, we review recent advances in three interconnected aspects of M/NPs research: biomolecular recognition, nano-enabled enrichment, and catalytic degradation, with particular emphasis on the central role of interfaces. Biomolecular recognition elements, including antibodies, peptides, aptamers, and molecularly imprinted materials, enable selective identification of plastics through interfacial pattern recognition. Nanomaterials such as magnetic nanoparticles, plasmonic nanostructures, metal–organic frameworks, and carbon-based materials further facilitate selective capture and signal amplification in complex matrices. Emerging degradation strategies, including engineered enzymes, enzyme–nanomaterial hybrids, nanozymes, single-atom nanozymes, and advanced oxidation processes, rely on interface-mediated adsorption, catalytic activation, and polymer transformation. Future research should therefore focus on elucidating plastic–bio–nano interfacial mechanisms and leveraging these insights to integrate molecular recognition, nano-enabled enrichment, and catalytic transformation within multifunctional platforms.

**Graphical abstract:**

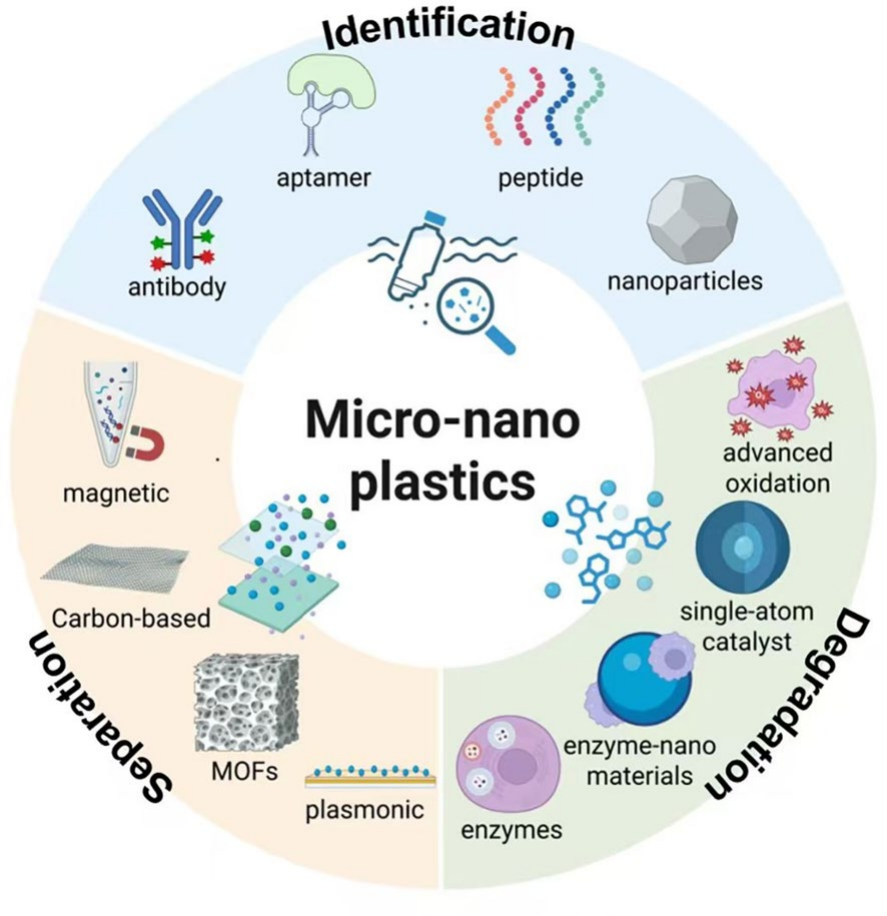

## Introduction

Micro-nano plastics (M/NPs) are ubiquitously detected across aquatic, terrestrial, and atmospheric systems [1], raising increasing concerns regarding their transport, transformation, and biological impacts [2]. Therefore, the detection and remediation of plastic contamination have been a hot research topic in recent years. A defining characteristic of M/NPs lies in their surface- and interface-dominated behavior [3] whereby interactions with natural organic matter, metal ions, microorganisms, and engineered materials govern their aggregation, mobility, aging, and bioavailability properties. With decreasing particle sizes, particularly in the nano-plastic regime, interfacial processes become increasingly dominant, complicating both detection and remediation under environmentally relevant conditions [4].

Advances in biotechnology and nanomaterials have opened new avenues for addressing the environmental challenges associated with M/NPs. Traditional approaches based on spectroscopy and chemical analysis often require extensive sample preparation and expensive instrumentation, making field deployment difficult. In addition, analytical and remediation technologies have largely evolved along separate paths: extraction and identification are typically pursued as standalone objectives, whereas degradation and removal are seldom incorporated into an integrated workflow [5]. Bridging these disconnected approaches will be essential for developing practical and scalable solutions for M/NP monitoring and remediation [6].

**Fig. 1:**
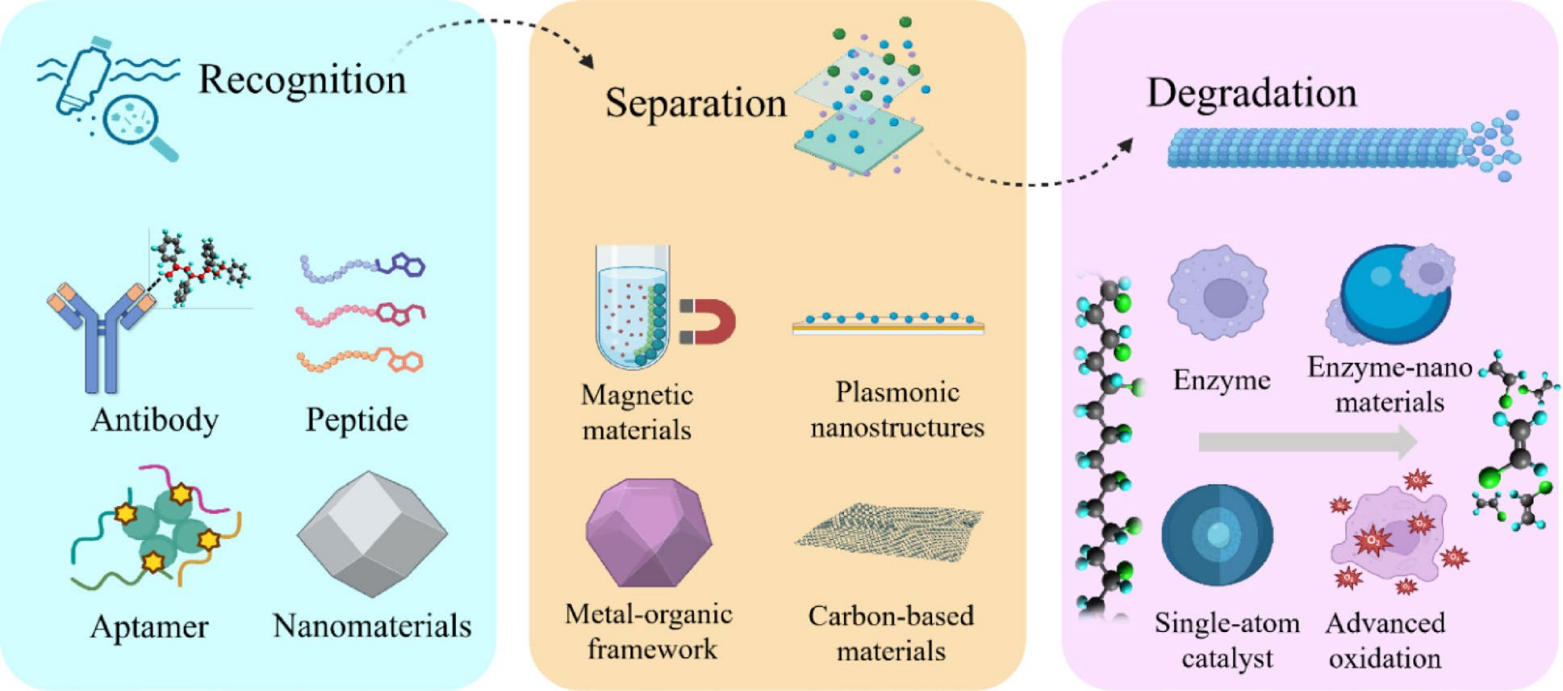
A scheme showing the processes of identification, enrichment and degradation of M/NPs based on bionanotechnology

Biological or biomimetic recognition elements, including antibodies [7], aptamers [8], peptides [9], and molecularly imprinted polymers [10], have been developed toward plastic materials or their associated additives, while a wide range of nanomaterials, such as magnetic particles, plasmonic nanostructures, metal–organic frameworks (MOFs), and carbon-based materials, enable enrichment, signal enhancement, or catalytic activities [11, 12]. In addition, these elements are increasingly combined to form integrated nanoscale systems, allowing M/NPs to be selectively captured from complex matrices and subsequently analyzed or transformed (Fig. 1).

Interfacial parameters play critical roles in the research of M/NPs (Table 1). Surface charge and hydrophobicity/hydrophilicity primarily govern the initial contact, adhesion, and aggregation of microplastics with recognition interfaces or environmental media. In contrast, the oxidation state and surface functional groups modify surface chemical characteristics, increase the availability of adsorption and reactive sites, and enhance the likelihood of subsequent chain scission, making them most directly involved in degradation. The eco-corona can redefine the surface identity of microplastics and therefore operates across identification, enrichment, and degradation. By comparison, crystallinity exerts a relatively indirect influence on identification and enrichment, but becomes a key structural factor limiting degradation efficiency by restricting the penetration of oxidants, enzymes, and microorganisms into the polymer matrix. These parameters are not static, as the surface states of environmental M/NPs continuously evolve due to aging, natural organic matter adsorption, and biofilm formation. Such dynamic changes can reduce identification efficiency by altering surface signatures [13], shift adsorption from hydrophobic-dominated to multi-mechanistic interactions [14], and exert dual effects on catalytic processes by simultaneously increasing reactive sites and masking them through corona or biofilm coverage [15].

**Table 1:** Interfacial controls on identification, enrichment, and degradation of M/NPs

Interfacial parameters	Identification	Enrichment	Degradation
Surface charge	Governs initial interactions for recognition [16]	Regulates electrostatic adsorption and hetero-aggregation [17]	Influences electron transfer at catalytic interfaces [18]
Hydrophilicity/ hydrophobicity	Determines surface compatibility and adhesion [16]	Drives hydrophobic partitioning and adsorption behavior [19]	Affects wetting and contact efficiency of enzymes/catalysts [20]
Surface functional groups	Alters surface characteristics and recognizability	Increases the availability of adsorption sites [21]	Provides active sites and facilitates chain scission [22]
Eco-corona	Redefines surface identity [16]	Modifies aggregation and deposition behavior [23]	Either promotes or inhibits degradation [24, 25]
Crystallinity	Indirect affects; surface characteristics	Controls molecular accessibility and diffusion [26]	Restricts penetration of oxidants/enzymes into the bulk [22]

In this review, we examine recent progress in molecular recognition strategies, nanomaterial-enabled enrichment and signal amplification, and emerging catalytic degradation pathways, including engineered enzymes, enzyme–nanomaterial hybrids, nanozymes, single-atom nanozymes, and advanced oxidation processes. Existing reviews on microplastic analysis and remediation have primarily examined analytical detection [27], adsorption-based removal [28], and catalytic degradation [22] as separate technological areas. However, the relationships among these processes and their potential integration into a unified workflow have received far less attention. In this review, we adopt a bionanotechnology- and interface-centric perspective that links biomolecular recognition, nanomaterial-enabled enrichment, and catalytic transformation into a coherent framework for M/NPs research. By integrating advances across these traditionally distinct fields, we identify shared interfacial mechanisms, highlight key knowledge gaps, and propose future directions toward scalable, nano-enabled platforms for the detection, separation, and remediation of M/NPs.

## Biomolecules for the recognition of M/NPs

M/NPs lack well-defined molecular epitopes, making them challenging targets for conventional molecular recognition systems. Consequently, most detection methods rely on spectroscopic analysis or physical separation, which often suffer from limited sensitivity, low throughput, and poor portability, particularly in complex matrices and at environmentally relevant concentrations [29]. Recent studies have shown, however, that despite the absence of specific functional groups, M/NPs possess distinct interfacial properties, including hydrophobicity, aromatic character, surface charge distribution, and morphology, that can be recognized by biomolecules through interfacial pattern-recognition mechanisms.

### Antibody-based recognition

**Fig. 2:**
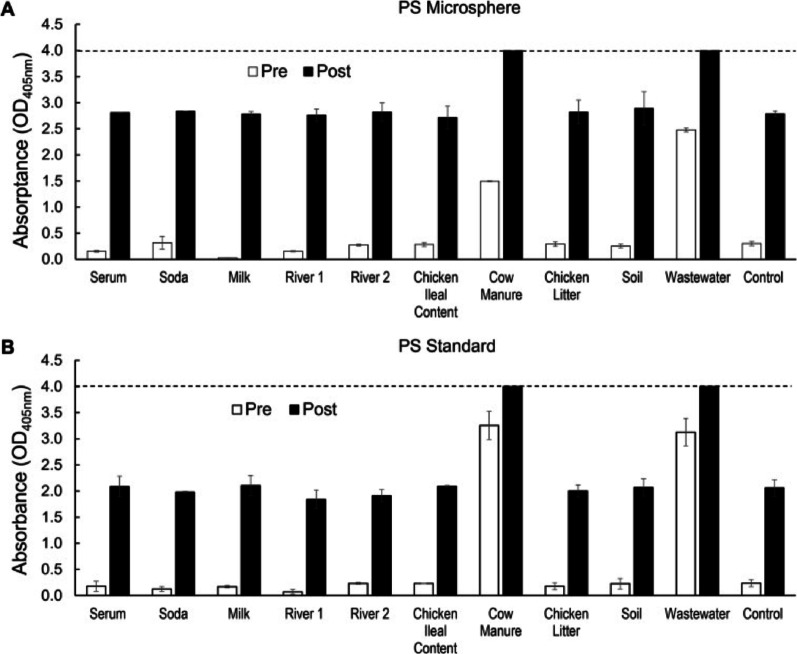
Matrix effects on PS antibody-based ELISA detection of polystyrene particles. **A** PS microspheres and **B** PS standard particles. Pre and Post denote pre-immune serum and PS-KLH-immunized antiserum, respectively. Cited from Reference [7] with permission. Copyright 2023 Elsevier

Although M/NPs are challenging targets for molecular recognition, efforts have been made to generate antibodies against them. For example, Cao et al. demonstrated that antibodies can be raised against the polymeric backbone of microplastics, providing evidence that synthetic polymer surfaces can be recognized with a degree of specificity by proteins [7]. In this study, polystyrene (PS) was used as a model plastic. By covalently conjugating nanoscale PS particles to a carrier protein, the authors overcame the inherently low immunogenicity of plastics and generated PS-selective polyclonal antibodies. These antibodies recognized PS particles across a range of sizes and molecular weights and retained binding activity in diverse environmental and biological matrices, including serum, beverages, river water, and solid samples (Fig. 2). While background signals remained low in most matrices, substantial interference was observed in organic-rich samples such as cattle manure and municipal wastewater, likely due to nonspecific adsorption or surface fouling. These results suggest that sample pretreatment may still be necessary for reliable detection in highly complex matrices.

However, the specificity of these antibodies remains incompletely characterized. The authors did not examine binding to other types of M/NPs, making it unclear whether the antibodies selectively recognize PS or exhibit broader adsorption to plastic surfaces. This distinction is particularly important because antibody adsorption onto plastics is a well-established phenomenon and is routinely exploited in immunoassays, where capture antibodies are immobilized on plastic plates through nonspecific adsorption. Therefore, rigorous discrimination between specific recognition and nonspecific surface adsorption is essential for validating antibodies against microplastics.

While the molecular basis of the recognition was not fully elucidated, instead of relying on discrete epitopes, recognition may arise from antibody responses to the collective physicochemical properties of plastic surfaces, such as hydrophobicity, morphology, and charge. Future studies should also examine the effects of surface aging, oxidation, and biofouling on recognition. Notably, this remains the only report of antibodies against M/NPs, and no follow-up studies have emerged since its publication in 2023. In contrast, most immunoassays target plastic-derived additives or degradation products, such as bisphenol A and plasticizers [30]. Although these approaches do not directly detect microplastic particles, they provide complementary information for exposure and risk assessment. Overall, this work demonstrates the feasibility of antibody-mediated microplastic recognition, but concerns regarding nonspecific adsorption of large protein molecules remain. Consequently, short peptides, with greater structural tunability and adaptability to material interfaces, are emerging as promising recognition elements that bridge biological recognition and interfacial engineering.

### Peptide-based interfacial binding

Compared to the limited studies on plastic-binding antibodies, a lot more work has been devoted to the design of peptides that can bind M/NPs [31]. By tuning the composition and arrangement of hydrophobic, aromatic, and charged residues, peptides may be designed to recognize distinct interfacial patterns on different plastic surfaces. Woo et al. demonstrated that engineered peptides can serve as effective recognition elements for microplastics, with highly hydrophobic peptide sequences capable of selectively binding common environmental plastics such as PS and polypropylene (PP) primarily through hydrophobic interactions [32]. Table 2 (for PS) and Table 3 (for PP) present the sequences, lengths and hydrophobicity of these plastic-binding peptides. This work mainly selected HWGMWSY as a PS binding peptide and MPAVMSSAQVPR as a PP binding peptide due to their suitable hydrophobicity and affinity. Peptide binding and selectivity were validated in simulated seawater conditions, supporting the environmental applicability of peptide-based microplastic detection in high-ionic-strength systems. However, a noticeable reduction in binding efficiency was observed for PS under saline conditions, which can be attributed to electrostatic screening of basic residues, disruption of π–π interactions, and decreased peptide solubility at high ionic strength, whereas PP binding remained largely unaffected due to its predominantly hydrophobic interaction mechanism. Furthermore, the inclusion of oxidized plastics, which better represent environmentally weathered microplastics [33], demonstrated that this approach remains effective under conditions relevant to natural environments. Overall, this study also indicates that peptide–plastic interactions are largely governed by the physicochemical properties of plastic surfaces rather than specific chemical structures.

Comparison of reported peptide sequences reveals no conserved motifs for either PS- or PP-binding peptides. Instead, substantial variation exists in sequence length, hydrophobicity, and amino acid composition, even among peptides targeting the same plastic type. This diversity suggests multiple binding modes and potentially a significant contribution from nonspecific adsorption.

**Table 2:** Reported PS-binding peptides

Sequence	Length	Hydrophobic residues (%)	Source
RAFIASRRIKRP [34]	12	33	Rationally designed
RAFIASRRIRRP [35]	12	33	Experimentally screened
RIIIRRIRR [35]	9	44	Experimentally screened
HWGMWSY [36]	7	57	Experimentally screened
KLWWMIRRW [37]	9	67	Rationally designed
LKKLLKLLKKLLKL	14	57	Rationally designed
KGLRGWREMISL [38]	12	42	Experimentally screened
TLHPAAD [39]	7	43	Experimentally screened
TSTASPTMQSKIR [32]	13	15	Rational design
KRNHWQRMHLSA [32]	12	23	Rational design
SHATPPQGLGPQ [32]	12	17	Rational design
PS QA Team [40]	12	/	Computational design
GAN 2 [9]	12	/	Computational design

**Table 3:** Reported PP-binding peptides

Sequence	Length	Hydrophobic residues (%)	Source
TSDIKSRSPHHR [32]	12	8.3	Rational design
HTONMRMYEPWF [32]	21	41.67	Rational design
LPPGSLA [32]	7	42.86	Rational design
MPAVMSSAOVPR [32]	12	50	Rational design
NOSFLPLDFPFR [32]	12	41.67	Rational design
SILSTMSPHGAT [32]	12	25	Rational design
SMKYSHSTAPAL [32]	12	41.67	Rational design
PS QA [40]	12	/	Computational design
NDLMFRRGLIFW [41]	12	/	Computational design
FWWQQIGGNRQF [41]	12	/	Computational design
SNMMFRRGLIHW [41]	12	/	Computational design
TAFMFRRGLIFW [41]	12	/	Computational design
GAN 2 [9]	12	/	Computational design

Early studies primarily relied on rational design and experimental screening to identify plastic-binding peptides, but recent efforts have increasingly shifted toward computational approaches driven by advances in artificial intelligence (AI), machine learning (ML), and the growing availability of sequence datasets [31, 42]. For example, a class of 12-mer multiplastic-binding peptides were designed using protein language model–guided generative adversarial networks. These peptide sequences typically exhibit amphiphilic structural characteristics, with hydrophobic amino acids (F, W, and L) enriched at the N-terminus and hydrophilic residues (R) at the C-terminus, enabling effective adsorption onto multiple plastic types, including PE, PP, and PET. However, validation was primarily computational: molecular dynamics simulations showed that GAN-designed peptides exhibited ~ 30% improved adsorption free energies compared to PepBD-Multi controls, with one sequence (GAN 2) showing stronger affinity for PE and PP than PET. No experimental selectivity data or quantitative fold differences were reported. Overall, compared to antibodies, peptides have advantages in stability, cost, and structural tunability, making them well suited for integration with nanomaterials and diverse signal transduction modalities in microplastics detection under complex environmental conditions.

These peptides have been integrated with nanomaterials to create sensitive sensing platforms. A common approach couples plastic-binding peptides with gold-based nanomaterials, where peptide–microplastic interactions are transduced into optical signals, including colorimetric responses, localized surface plasmon resonance (LSPR) shifts, and enhanced surface-enhanced Raman scattering (SERS). Such signal amplification strategies substantially improve detection sensitivity. For example, in a peptide-functionalized Fe_3_O_4_@Au composite nanoparticle platform for PS, the peptide provides selective capture, the Fe_3_O_4_ core enables magnetic enrichment and separation, and the gold shell generates the UV–vis optical response. This system showed a linear response from 0 to 80 pg·mL^− 1^, with an LOD of 0.074 pg·mL^− 1^ and 3.1% RSD at 80 pg·mL^− 1^; it also produced a detectable signal within 10 min and reached stable readout in 20 min. In spiked bottled water, salt, and milk samples, the recoveries ranged from 89.15–110.91%, confirming the feasibility of an integrated workflow that combines recognition, separation, and detection in a single platform [43]. Similarly, peptide–gold nanoparticle systems have been leveraged to develop rapid colorimetric or plasmonic sensors for straightforward, visual detection of microplastics [44, 45]. Notably, the selectivity of these systems is dictated by the intrinsic affinity of peptides toward specific polymer types. For example, LCI and TA2 preferentially bind PP and PS, respectively, enabling selective detection in the presence of other common plastics (e.g., PVC, PET, and PE). However, their applicability to diverse microplastics remains limited, underscoring the need for expanded peptide libraries.

Beyond optical platforms, peptides have also shown strong compatibility with electrochemical sensing systems. By immobilizing plastic-binding peptides onto electrode surfaces, adsorption of microplastics can modulate interfacial charge transfer and/or alter the electrical double-layer structure, leading to pronounced changes in electrochemical impedance or current responses. Studies combining fluorescently labeled peptides with electrochemical impedance spectroscopy (EIS) have demonstrated quantitative analysis of PS microplastics under different water conditions, with resolvable response characteristics toward particle size, concentration, and environmental ionic strength [46]. Here, fluorescent labeling is mainly used to validate peptide–microplastic interactions, while the electrochemical signal is generated from impedance changes at the electrode interface. Peptide–EIS platform enabled detection over 50 ppb–20 ppm, with a LOD of 50 ppb in pure and tap water and 400 ppb in saline/seawater, while requiring only 5 min for peptide binding and signal acquisition. Such approaches provide an important technological route for peptide-mediated, portable, and on-site detection of microplastics.

At the device level, peptides have also been incorporated into microstructured and wearable sensing platforms to enable in situ or localized sampling. For example, peptide-functionalized microneedle arrays can selectively capture microplastic particles directly from environmental media or complex samples, acting as microstructured collectors that enable the efficient capture and enrichment of microplastics for subsequent Raman or SERS-based analysis. Such designs fully exploit the interfacial recognition capabilities of peptides while overcoming the reliance of conventional methods on extensive sample pretreatment and bulky instrumentation. These advances highlight the considerable potential of peptide-based systems for on-site detection and rapid screening of microplastics [47].

Another line of research achieves visual labeling of microplastics by directly anchoring fluorescent proteins onto plastic surfaces. In this strategy, plastic-binding peptides serve as anchoring motifs, and peptide–fluorescent protein fusion constructs are engineered to enable GFP to stably adsorb onto plastic substrates, thereby allowing direct fluorescent tagging of microplastic particles. By combining the interfacial recognition capability of peptides with the high signal-to-noise optical properties of fluorescent proteins, this approach endows plastic particles with readily distinguishable fluorescence signals for microscopy-based observation or flow-based analysis. Quantitative analysis further revealed nanomolar binding affinities, with representative peptide–protein fusions exhibiting apparent dissociation constants of ~ 176 nM for PP and ~ 260 nM for PS. Notably, this strategy conceptually demonstrates the programmable modularity of protein-based systems: recognition and reporting functions can be rationally integrated within a single molecular design to operate synergistically [48]. However, control proteins without peptide anchors also showed comparable adsorption, and peptide incorporation improved binding only modestly (2.9 to 4.5-fold), indicating substantial nonspecific interactions.

### Cell–based M/NPs detection

Living organisms have also been exploited to detect M/NPs. For example, a *Pseudomonas aeruginosa* whole-cell biosensor employs the cdrA promoter, associated with surface adhesion and biofilm formation, to drive GFP expression upon exposure to microplastics. This system achieves a detection limit of 1 ng/mL within 3 h and shows good selectivity, with negligible responses to non-plastic particles such as sand, glass, or alginate. This approach does not rely on direct binding or specific recognition between GFP and microplastic particles but instead is based on particle induced cellular physiological responses such as surface adhesion, stress responses, or metabolic alterations. This strategy is particularly meaningful for environmental monitoring and ecotoxicological investigations, as it also assesses biological effects of microplastics [49].

### DNA-based recognition at plastic interfaces

In addition to proteins, nucleic acids represent another important class of recognition elements. Their programmable sequences and structures enable adaptive interactions with heterogeneous plastic interfaces. Furthermore, DNA oligonucleotides can be synthesized inexpensively, facilitating systematic studies of structure–binding relationships and recognition mechanisms.

#### DNA–microplastic interfacial interactions as a recognition basis

DNA sequences that selectively bind target molecules are known as aptamers. Before applying aptamers to microplastic detection, the interactions between DNA and plastic surfaces must first be understood. As a negatively charged, flexible, and structurally programmable polymer, DNA displays diverse and tunable adsorption behaviors at material interfaces, offering opportunities for nucleic acid–based recognition of M/NPs. DNA can adsorb onto plastics through multiple noncovalent mechanisms, including electrostatic interactions, hydrophobic interactions, π–π interactions, and van der Waals forces [4].

We found that typical linear DNA oligonucleotides and spherical DNA nanostructures (DNA densely functionalized on gold nanoparticles [50]) display distinct adsorption behaviors on microplastics. Compared with flexible linear DNA, spherical DNA owing to its high local DNA density and conformational confinement exhibits more stable and reproducible adsorption upon contacting microplastics, while also providing stronger discriminative capability toward different polymer types and surface states [51]. Furthermore, DNA–MP interactions have been exploited as an inverse readout to characterize the surface physicochemical properties of microplastics such as wettability [52].

#### Recognition of microplastics by DNA aptamers

**Fig. 3:**
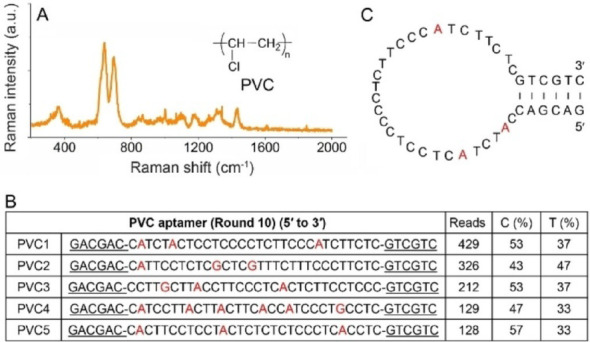
**A** Raman spectroscopy characterization of the PVC plastic used for aptamer selection. **B** Alignment of the top five PVC binding aptamers and their C/T base contents. **C** The secondary structure of the PVC1 aptamer, which has no stable structure in the loop region to maximize the loop interactions with the microplastic surface. Cited from Reference 8 with permission. Copyright 2024 Wiley

Aptamers have been obtained to bind to various small molecules [53, 54], proteins [53, 54], and even cells and tissues [55]. Our group selected DNA aptamers that can bind to PS and PVC microplastics [8]. Figure 3B shows the alignment of the five most abundant sequences enriched after 10 rounds of selection, while Fig. 3C schematically illustrates the secondary structure of the most abundant aptamer. The fact that the same sequence evolved for both PS and PVC suggested that aptamer–microplastic binding does not depend on specific chemical functional groups. These T/C-rich sequences have little internal secondary structures and thus allow them to maximize contact with plastic surfaces. This mechanism is highly consistent with the interfacial pattern-recognition concept.

Building on these fundamental studies, the engineering applications of aptamers have expanded rapidly. A representative example is the label-free electrochemical CRISPR-MP platform developed by Shi et al. [56]. In this work, a cascade architecture of plastic-binding aptamer–nucleic acid reaction network–CRISPR cleavage–electrochemical readout was constructed to transduce microplastic recognition events into amplifiable electrochemical signal changes. The aptamer is immobilized on the electrode surface, and binding of microplastic particles induces perturbations in interfacial charge transfer and the electrical double layer, yielding a measurable electrochemical impedance response. The reported limits of detection for PVC and PS were 37 ng/mL and 45 ng/mL, respectively, and spike–recovery tests in tap water and river water produced recoveries of ~ 97.6–103.7%, indicating compatibility with complex matrices. In addition, the authors evaluated the impacts of aging and temperature on binding and signal transduction: heat-aged PVC/PS generated signal responses comparable to those of unaged samples, whereas larger signal changes were observed at 4 °C, suggesting temperature dependence in microplastic–aptamer binding and/or downstream activation processes.

### Low-specificity but high-throughput recognition and labeling approaches

Beyond highly selective recognition elements, many rapid screening and visualization methods for M/NPs rely on nonspecific interfacial interactions rather than well-defined binding sites. In these approaches, nanomaterial probes transduce processes such as hydrophobic adsorption, surface coating, hydrogen bonding, and aggregation into measurable optical or electrical signals, enabling rapid detection and semi-quantitative analysis. Although less selective, these methods offer operational simplicity, high throughput, and suitability for preliminary screening and imaging applications.

**Fig. 4:**
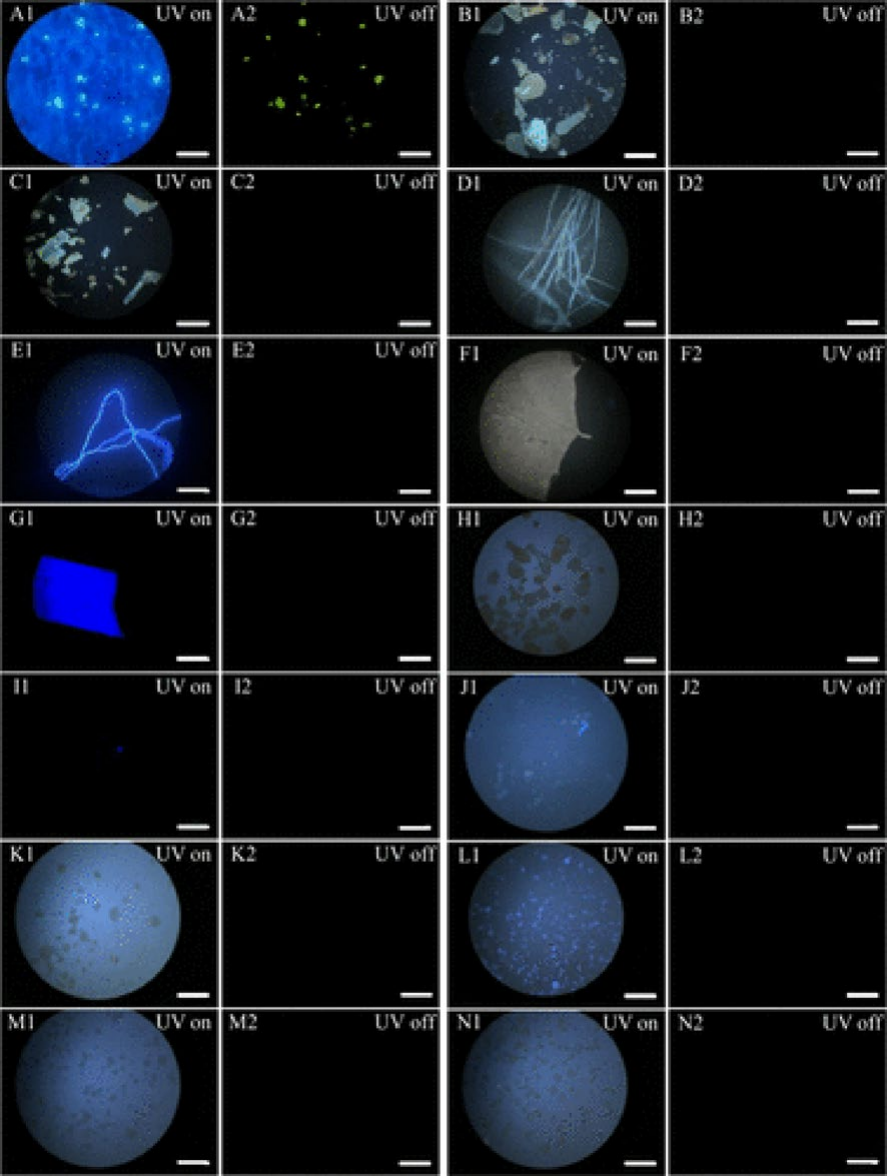
Micrographs of microplastics after being stained with NDCNQDs. Cited from Reference [58] with permission. Copyright 2025 ACS Publications

Small-molecule dyes (e.g., Nile Red [57]) remain widely used for hydrophobic staining, but they are prone to nonspecific labeling and autofluorescence interference in complex environmental matrices, which can cause false positives. To improve robustness, quantum dot–based probes (including carbon nitride/carbon dots) have been tested, offering higher photostability and, in some cases, time-resolved emission that suppresses short-lived background fluorescence and improves signal-to-noise ratios (Fig. 4). Naphthalene-doped carbon nitride quantum dots (NDCNQDs) contain abundant –NH, –OH, and C = O functional groups, which enable selective adsorption onto polyamide (PA) microplastic via hydrogen bonding interactions with amide groups in the polymer backbone, rather than nonspecific hydrophobic interactions. Competitive experiments using urea (a hydrogen-bond disruptor) significantly suppressed the signal, confirming that hydrogen bonding dominates the recognition mechanism. In validation using real samples, this approach achieved favorable recoveries in complex environmental matrices such as pond water, and could be coupled with Raman spectroscopy to further confirm the polymer composition of detected microplastics [58].

Upconversion nanomaterials, featuring NIR excitation and visible emission, further reduce autofluorescence and are therefore attractive for imaging, tracing, and uptake/biodistribution studies [59]. Carbon-based nanomaterials can also function as adsorption/aggregation-responsive sensors, often via fluorescence quenching [60, 61]; when combined with conductive matrices, dual-mode fluorescence–resistance readouts provide complementary validation under field-relevant conditions [62]. Overall, these strategies prioritize simplicity, speed, and throughput, but their mechanisms generally lack polymer specificity and remain sensitive to matrix effects.

### Comparative performance and application positioning of recognition units

Although the above recognition units have all demonstrated feasibility in M/NPs detection, pronounced differences exist among them in terms of recognition mechanisms, applicable plastic types, and detection scales. Table 4 provides a systematic comparison of the performance of the major recognition elements reported to date. These strategies are not mutually substitutive in their application positioning; rather, they exhibit clear complementarity across different detection scenarios and analytical objectives.

**Table 4:** Comparison of different recognition elements for M/NPs detection

Recognition unit	Types of M/NPs	Size	Detection mode	Sample	LOD	Ref.
aptamer	PS, PVC	µm	fluorescence	waste water	0.5 mg; 0.6 mg	[8]
aptamer	PS, PVC	µm	electrochemistry	tap water; river water	45 ppb; 37 ppb	[56]
antibody	PS	nm, µm	/	/	/	[7]
peptide	PE, PP, PET	µm	/	/	/	[9]
peptide	PET	µm	colorimetry	/	/	[44]
peptide	PP, PS	µm	colorimetry	lake water	/	[45]
peptide	/	nm	fluorescence	/	/	[42]
peptide	PS	nm	LSPR	lake water	/	[63]
peptide	PP, PS	µm	SERS	organisms	/	[47]
peptide	PS	µm	fluorescence, electrochemistry	tap water	50 ppb	[46]
peptide	PP, PS	µm	/	organisms	/	[32]
peptide	PS	nm, µm	UV	bottled water, table salt, milk	0.074 pg/mL	[43]
GFP	PS	µm	fluorescence	seawater	1 ppb	[49]
Co-CQDs	PS	µm	fluorescence	/	0.4 mg/L	[64]
NDCNQDs	PA	µm	fluorescence	stream water, sediment, dusts	/	[58]
EG-CQDs	PET	µm	fluorescence	bottled water	0.15 ppm	[61]
S-CNPs/PPy	PE	µm	fluorescence, electrochemistry	seawater and river water	/	[62]
SFGCDs	masks, cosmetic cleaners	µm	fluorescence	water	6.3 mg/L	[60]

Environmental plastics are typically coated with bacteria, grease, dissolved organic matter, and other contaminants. Consequently, biomolecular ligands designed to recognize pristine plastic surfaces may have limited access to the underlying polymers, making sample pretreatment, such as surfactant washing, important for reliable detection. In contrast, nonspecific probes, including small-molecule dyes and carbon- or quantum-dot-based materials, offer operational simplicity and high throughput but are more susceptible to matrix interference because their signals arise from general interfacial interactions. These considerations suggest that future M/NPs detection platforms will likely integrate multiple recognition strategies with nanomaterial-enabled signal amplification, rather than relying on a single universal ligand. Combining affinity ligands such as aptamers or peptides with spectroscopic techniques such as Raman spectroscopy may provide dual-layer specificity [47]: affinity ligands can enrich and separate plastic particles from nonplastic materials and even major types of plastics, while Raman spectroscopy can identify the polymer type.

## Nano-enabled and enhanced adsorption materials for M/NPs

Beyond detection, the enrichment and concentration of M/NPs are critical for both analysis and remediation. Challenges associated with complex environmental matrices, low plastic concentrations, and nanoscale particles have motivated the development of advanced adsorption materials for M/NPs capture. At the same time, nanomaterials can serve as photocatalysts, electrocatalysts, or nanozymes to promote plastic degradation, bridging the gap between detection and remediation and enabling more integrated strategies for M/NPs management.

### Magnetic nanomaterials for rapid extraction

Magnetic nanoparticles, particularly superparamagnetic Fe_3_O_4_ and γ-Fe_2_O_3_, enable rapid separation from liquids under an applied magnetic field, serving as effective, low-cost, recyclable carriers for M/NPs capture. Surface modifications with hydrophobic chains, chitosan, or molecularly imprinted polymers can further tune their interactions with M/NPs. Our group found that unmodified Fe_3_O_4_ nanoparticles showed superior microplastic adsorption capacity compared to hydrophobically modified Fe_3_O_4_ [65], which might be due to better dispersion of the unmodified nanoparticles in aqueous solutions.

**Fig. 5:**
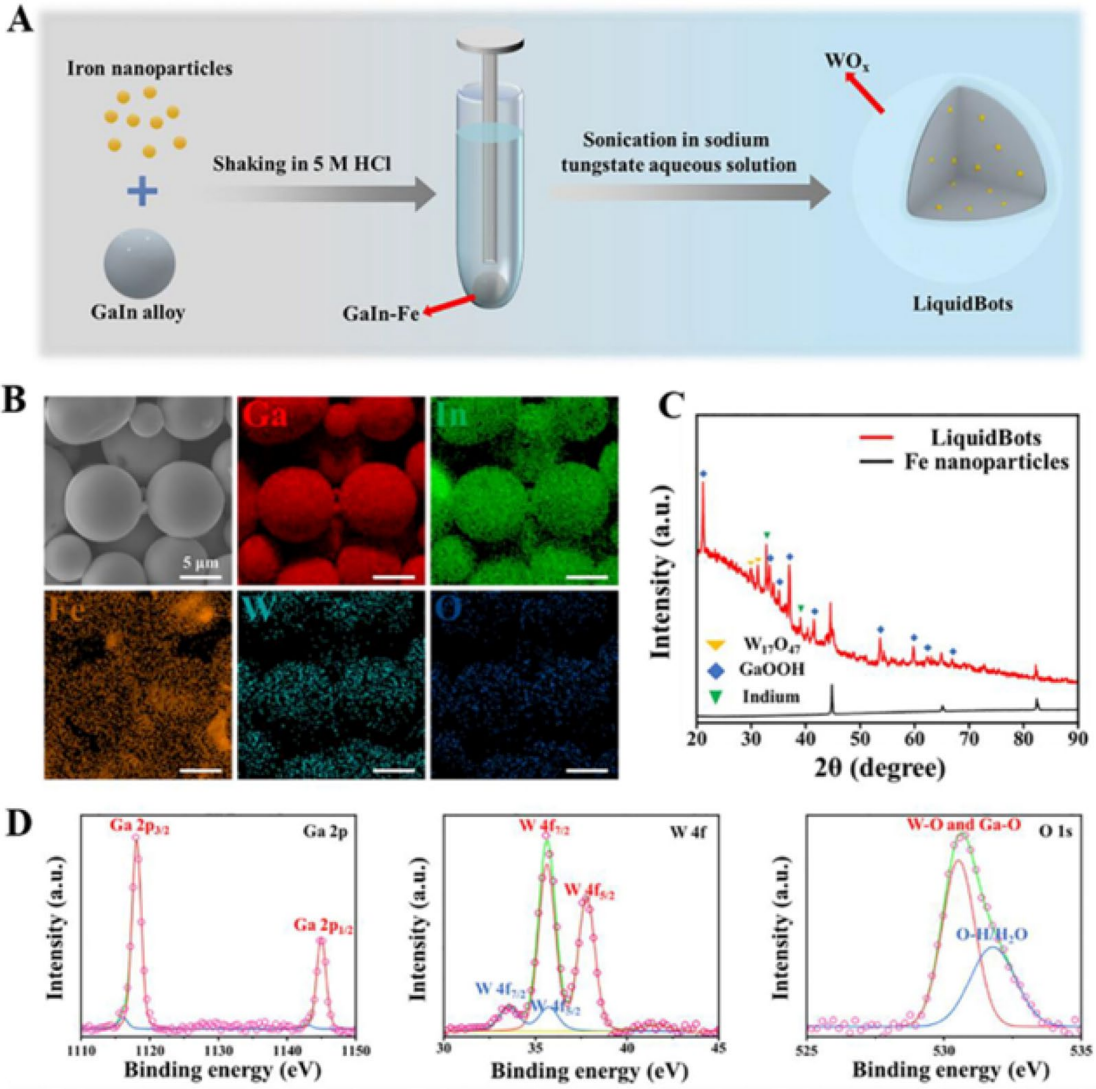
Synthesis and characterization of LiquidBots. **A** Schematic representation of the synthesis process. Reproduced (Adapted) under the terms of the Creative Commons CC-BY license. Copyright 2024, Wiley-VCH. **B** SEM images with corresponding EDX mapping of LiquidBots. Scale bar: 5 μm. **C** XRD patterns of LiquidBots and Fe nanoparticles. **D** Ga 2p, W 4f, and O 1s spectra of LiquidBots. Cited from Reference [66] (open access article)

Recent advances include intelligent responsive systems, such as light- or magnetically-driven micro/nanorobots, which enable active capture and directional movement of M/NPs under external stimuli [66–68]. Figure 5 illustrates the fabrication and characterization of light/magnetically-driven liquid metal-based microrobots. Light-driven micro/nanorobots (e.g., geometry-programmed silicon microrobots, MoS_2_/Fe_2_O_3_ piranha micromotors, and reconfigurable self-assembling liquid metal LiquidBots) achieve autonomous motion via photocatalytic/photoelectrochemical effects and are often integrated with magnetic components for precise maneuvering. In the context of microplastic capture, they can achieve removal rates exceeding 95% with adsorption capacities as high as 3060 mg·g^− 1^, while simultaneously enabling the gradual degradation of microplastics through reactive oxygen species (ROS)-mediated photocatalysis [69, 70]. By integrating recognition, capture, and degradation, these systems provide multifunctional platforms for plastic pollution management. However, improvements in motion control, targeting accuracy, and long-term stability are still needed for practical applications.

From an application standpoint, incorporating magnetic materials into membrane filtration and sedimentation processes can enhance M/NPs removal efficiency while mitigating membrane fouling, thereby improving the overall performance of water treatment systems [71–73]. These hybrid approaches show potential in wastewater treatment, sludge processing, and liquid food purification [74]. Nevertheless, performance can decrease in high-salinity water or in the presence of natural organic matter, and long-term cycling stability requires further improvement for large-scale use [75].

### Plasmonic nanostructures for signal-amplified adsorption

While magnetic separation excels at concentrating M/NPs from large volumes, the unambiguous identification of plastic types remains a challenge. Surface-enhanced Raman scattering (SERS) leverages metallic nanostructures to significantly amplify Raman signals, enabling highly sensitive detection of M/NPs. To achieve ultra-low detection limits, a primary focus in SERS substrate design is the creation of intense electromagnetic ‘hot spots’ at anisotropic features like sharp tips. Anisotropic nanostructures, such as nanostars and nanourchins, are particularly effective in this regard [76–78]. Their sharp tips concentrate local electromagnetic fields, enabling the detection of common polymers at trace concentrations (e.g., down to ng·mL^− 1^ levels). Other strategies, including film-based substrates for analyte enrichment and hybrid structures for improved stability, further contribute to practical SERS applications [62, 79, 80].

Beyond laboratory-scale substrate design, SERS technology is also evolving toward portability and intelligence [81]. To further improve practicality, portable SERS systems incorporating miniaturized Raman spectrometers and fiber-optic probes facilitate field-deployable M/NPs monitoring. Additionally, coupling SERS with machine learning algorithms allows for accurate classification and semi-quantification of mixed plastic samples, overcoming limitations of traditional spectral analysis [82, 83]. In our opinion, SERS is more attractive for nanoplastics than for microplastics, since the SERS effect is only sensitive to species in close contact with metal nanostructures.

### Porous frameworks for size-selective and surface-adaptive adsorption

MOFs have emerged as promising porous materials for M/NPs remediation due to their high surface areas, tunable pore sizes, and customizable surface chemistries. By selecting appropriate metal nodes and organic linkers, the pore environment and surface functionality of MOFs can be precisely engineered to enhance adsorption selectivity toward diverse M/NPs through hydrophobic interactions, hydrogen bonding, and π-π stacking, often exhibiting molecular sieving effects even in complex environmental matrices [84, 85].

Composite MOF materials, including magnetic variants (e.g., Fe_3_O_4_@ZIF-8) or defect-engineered frameworks (e.g., Zn-Co-ZIF), improve practicality by facilitating material recovery and enhancing stability under real-world conditions. These materials typically exhibit high removal efficiencies (70–99.9%) for common plastics such as PS, PE and PP, with the possibility of regeneration and reuse across multiple cycles [86]. Continued research focuses on improving structural robustness in high-salinity environments and scaling up production for practical water treatment applications [87, 88].

### Carbon-based nanomaterials as multifunctional adsorption scaffolds

Carbon-based nanomaterials, including graphene oxide (GO), carbon nanotubes (CNTs), and carbon quantum dots (CQDs), serve not only as a cost-effective and stable alternative to noble metal-based SERS substrates, albeit typically with lower enhancement factors, but also as versatile platforms for M/NPs removal and detection owing to their tunable surface chemistry, high specific surface area, and robust stability [89, 90]. These materials interact with M/NPs through hydrophobic effects, π-π stacking, and electrostatic interactions, enabling efficient capture even in complex matrices. For instance, magnetic CNTs functionalized with hydrophobic or π-rich moieties achieve rapid adsorption of PE and PP, with removal efficiencies exceeding 90% within minutes [91]. Similarly, amino-modified carbon fibers exhibit enhanced electrostatic attraction toward negatively charged polystyrene nanoplastics, facilitating rapid removal from seawater and wastewater. For detection, carbon nanomaterials amplify signals in electrochemical and optical sensors. GO-based electrochemical platforms detect M/NPs via conductivity changes induced by adsorption, while CQDs enable fluorescence-based sensing through quenching mechanisms. Hybrid composites (e.g., Fe_3_O_4_/GO) further integrate magnetic separation with sensing, allowing simultaneous enrichment and quantification of M/NPs [90].

## Pathways for degradation of M/NPs

In this final section, we discuss degradation of M/NPs, which primarily relies on the cleavage of chemical bonds within polymer chains, the introduction of oxygen-containing functional groups into the polymer backbone to facilitate fragmentation, or the subsequent transformation of polymer chains and their intermediates into low-molecular-weight carbonaceous compounds via biological and chemical processes. Current degradation strategies can be broadly categorized into four main types:

(i) **Enzymatic degradation systems**: Polymer chains are oxidized and hydrolyzed into oligomers and monomers through the binding interactions between enzymes and their substrates.
(ii) **Enzyme–nanomaterial hybrid systems**: By coupling enzymes with nanomaterials, the stability of the degradation system and its catalytic efficiency can be enhanced.
(iii) **Inorganic nanocatalytic systems** (including nanozymes and single-atom nanozymes): These systems enable efficient degradation of microplastics at the nanoscale.
(iv) **Photocatalytic and Fenton-like advanced oxidation processes (AOPs)**: Under strong oxidative conditions, ROS attack polymer chains, thereby inducing the degradation of microplastics.

### Biocatalytic depolymerization of microplastics by native and engineered enzymes

Current enzymatic degradation studies have mainly focused on PET due to its hydrolyzable ester bonds in its backbone. Multiple enzymes capable of degrading PET have been identified within this system, including esterases, lipases, hydrolases, and keratinases. A landmark advance in this field was the identification of *Ideonella sakaiensis* and its PETase by Yoshida and co-workers. Compared with enzymes such as LCC, TfH, and HiC, PETase showed higher substrate specificity toward PET, highlighting the existence of naturally occurring microbial systems capable of PET depolymerization [92]. In-depth investigations of PETase have elucidated its catalytic mechanism at the molecular level, demonstrating that the core catalytic architecture of PETase relies on a serine (Ser)–histidine (His)–aspartate (Asp) catalytic triad located at the protein active site. PETase binds the substrate via a shallow cleft-like binding pocket and, through nucleophilic attack mediated by the catalytic triad, cleaves the ester bonds along the polymer backbone. Consequently, long-chain PET is initially hydrolyzed into mono(2-hydroxyethyl) terephthalate (MHET), bis(2-hydroxyethyl) terephthalate (BHET), and terephthalic acid (TPA). Subsequently, MHETase hydrolyzes MHET into TPA and ethylene glycol (EG), after which TPA is further assimilated into intracellular metabolic pathways, with a fraction ultimately undergoing complete mineralization to CO_2_ [93].

Although naturally occurring PET-degrading enzymes exhibit intrinsic catalytic activity, they generally suffer from poor thermal stability and limited degradation efficiency toward highly crystalline PET, thereby constraining their practical applicability in practical settings [94, 95]. Consequently, protein engineering has emerged as a central strategy for enhancing catalytic efficiency and stability under specific application conditions. Researchers have employed a combination of directed evolution, site-saturation mutagenesis, random mutagenesis, and rational design to optimize and tailor these enzymes [96, 97]. For example, Son and colleagues constructed a PETase variant through rational design, which exhibited a degradation activity approximately 14-fold higher than that of the wild-type enzyme [98]. In addition, the relevant studies have introduced disulfide bonds, salt bridges, or glycosylation modifications into the key flexible regions of enzymes, such as Cut190, to increase their melting temperature by 20–30 °C, thereby enabling these enzymes to maintain activity for extended periods at elevated temperatures [96]. For highly crystalline PET, TurboPETase designed through the integration of protein language models and molecular force-field algorithms has been demonstrated to efficiently degrade untreated highly crystalline PET films and powders [99]. At present, the commercialization of novel enzymes for PET degradation is advancing rapidly. For instance, companies such as Carbios are actively moving toward the establishment of commercial-scale facilities, and the commercialization experience gained from PET depolymerization may provide valuable insights for the development of degradation strategies for other plastics.

However, when moving from PET, which contains hydrolyzable ester linkages, to plastics with C–C backbones such as PE, PP, and PS, enzymatic degradation becomes far more challenging. The high bond dissociation energy, chemical inertness, and lack of surface heteroatoms make these polymers highly resistant to direct enzymatic hydrolysis [22, 100]. At present, the biodegradation of such C–C backbone plastics primarily relies on a slow and complex fungal enzymatic pathway. This process is typically initiated by oxidative enzymes, such as laccases and peroxidases, which introduce oxidative modifications that weaken polymer hydrophobicity, promote chain oxidation, and reduce molecular weight. The partially oxidized intermediates can subsequently be recognized and further attacked by the broader fungal enzymatic system [101–103]. However, this oxidative process remains highly inefficient, and experimental validation of its detailed catalytic cycle and key reaction intermediates is still at an early stage. Therefore, such enzymatic degradation processes currently remain largely at the stage of preliminary modification and partial degradation, and are still far from achieving complete degradation.

### Interface-enhanced enzymatic degradation through nanomaterial coupling

The small size and high specific surface area, and abundant porosity of nanomaterials enable effective interactions with microplastics [104]. The integration of enzymes with functional nanomaterials has emerged as a highly promising research direction for microplastic degradation. Depending on the intrinsic properties and functionalities of the nanocarriers, two major strategies have been developed: carrier-mediated enzyme immobilization and synergistic catalysis.

As enzyme carriers, Fe_3_O_4_ nanoparticles have attracted considerable attention due to their excellent biocompatibility, high specific surface area, and unique magnetic responsiveness [22]. For example, PETase can be directionally immobilized onto the surface of superparamagnetic Fe_3_O_4_ nanoparticles via affinity strategies such as His-tag anchoring. This immobilization retains approximately 50% of the enzymatic activity after 10 catalytic cycles, significantly enhancing enzyme stability. Moreover, the catalyst can be rapidly recovered under an external magnetic field for recyclability [105]. In addition, Hirata and colleagues immobilized a genetically engineered thermostable cutinase mutant onto superparamagnetic iron oxide nanoparticles (SPIONs) [106]. This combination of genetic modification and immobilization enabled long-term stability at 70 °C, while simultaneously increasing the monomer production by 6-fold. However, its degradation products remained at the intermediate stage, namely TPA, MHET, and BHET, and complete mineralization was not achieved. Beyond Fe_3_O_4_, other inorganic oxides such as SiO_2_ have also been explored as potential supports due to their high physicochemical stability and facile surface functionalization [107].

MOFs, owing to their exceptionally high specific surface area, tunable pore architectures, and diverse coordination environments, offer broad application prospects for enzyme immobilization and catalytic enhancement [108]. Encapsulation of enzymes within the nanoporous channels of MOFs not only protects them from external environmental stressors, but also modulates enzyme conformation through spatial confinement effects, which may further activate or enhance catalytic performance [109, 110]. Notably, certain MOF materials, such as ZIF-8 and UiO-66, have exhibited remarkable catalytic activity and recyclability in PET degradation and related reactions [108, 111]. However, such encapsulation may hinder the accessibility of the enzymes to microplastics.

### Polymer scission by nanozymes

Emerging biomimetic catalysts, particularly nanozymes and single-atom nanozymes (SAN), show great promise for microplastic degradation. Current research on nanozymes primarily focuses on developing nanozymes with peroxidase-like (POD-like) and oxidase-like (OXD-like) activities. These nanozymes offer several advantages, including high catalytic activity, enhanced stability, and recyclability [112, 113]. These nanozymes are capable of activating H_2_O_2_ or oxygen under mild conditions, generating reactive ROS, such as ·OH, which subsequently attack the inert carbon-carbon or carbon-nitrogen bonds on the surface of microplastics [114]. For example, hydrophilic bare Fe_3_O_4_ magnetic nanoparticle aggregates, leveraging their intrinsic superparamagnetism and peroxidase-like activity, enable the adsorption, separation, and catalytic degradation of microplastics. Under high-temperature conditions, they can catalyze the degradation of adsorbed microplastics, achieving degradation efficiencies close to 100% [65]. In another study, Zhang et al. employed nano-Fe_3_O_4_ as a peroxidase-like nanozyme to activate H_2_O_2_ through Fe^2+^/Fe^3+^ redox cycling, thereby enabling sustained ·OH generation and C–C bond cleavage along the backbone of PE microplastics. Under optimized conditions, this system achieved a 40.1% mass loss of PE microplastics within 7 h, accompanied by the formation of hydrocarbon and oxygenated products, including alkanes, carboxylic acids, alcohols, and esters. These findings demonstrate that Fe_3_O_4_ nanozymes can not only promote PE microplastic degradation under relatively mild, sub-melting-point conditions, but also facilitate their conversion into value-added chemical feedstocks with potential resource-recovery applications [115].

Beyond ROS-mediated nanozyme systems, hydrolase-mimicking nanozymes have recently emerged as an alternative strategy for polyester degradation. Wan et al. developed a thermophilic MgAl layered double oxide nanozyme with a memory effect, which mimics PET hydrolases by activating water molecules at Lewis basic sites to generate OH^−^ species for ester-bond cleavage. In contrast to peroxidase-like nanozymes that usually rely on ·OH driven oxidation, this system promoted hydrolytic depolymerization of PET powders, PET films, and other polyester substrates under mild alkaline conditions, while retaining high catalytic activity after repeated cycles. This work expands nanozyme-mediated microplastic degradation from oxidative chain scission toward enzyme-like hydrolytic depolymerization [116].

SANs achieve the maximization of atomic utilization by uniformly dispersing metal active sites at the atomic level on the surface of supports. Due to their unique electronic structures and coordination environments, SANs exhibit exceptional activity and selectivity [117], achieving efficient oxidation under thermal, electrical, or optical driving forces. A representative example is the SAN developed by Wang et al., which consists of a hierarchical porous carbon nitride support loaded with iron [118]. The Fe-N_4_ coordination structure, featuring an ordered and interconnected hierarchical porous architecture, facilitates the proximity of reactants to the active sites. Under mild conditions, it activates ROS to attack and activate the C-H bonds of microplastics, thereby initiating the oxidation and cleavage of polymer chains.

### Advanced oxidation pathways for deep polymer breakdown

In recent years, photocatalysis has gained widespread attention due to its sustainability and relatively low environmental impact [119]. Ultraviolet (UV) light-driven photocatalytic nanomaterials have been tested for the degradation of microplastics. Under UV irradiation, photocatalysts can effectively degrade a variety of common microplastics, including PE [120], PP [121], PS [122], and PET [123]. When a semiconductor is photoexcited with energy greater than its band gap, electrons in the valence band absorb the energy of photons and break free from atomic bindings, transitioning from the valence band to the conduction band. The direct result is the generation of a free electron (e^−^ _CB_) and a positively charged hole (h^+^ _VB_). Both e^−^ _CB_ and h^+^ _VB_ react with O_2_ and H_2_O molecules on the surface of the semiconductor to produce reactive oxygen species (ROS) such as O_2_·⁻ and ·OH. These ROS species attack microplastics, causing polymer chain cleavage [124, 125]. The degradation process is typically accompanied by an increase in the hydrophilicity of the plastic surface, the formation of oxygen-containing functional groups such as carbonyl and hydroxyl groups, and ultimately the breakdown into small organic molecules, which can even be completely mineralized to CO_2_ and H_2_O [126–128]. The specific process is photocatalytic degradation of microplastic follows a sequential radical-mediated pathway: photoexcited electrons in the e-CB first react with dissolved O_2_ to generate O_2_·⁻, which then react with H_2_O to form ·OOH and OH-, ·OOH radicals subsequently disproportionate to produce O_2_ and H_2_O_2_, and the resulting H_2_O_2_ is further activated by hν to yield highly reactive ·OH, Finally, both ·OH and O_2_·⁻ radicals attack the polymer chains, inducing chain scission and oxidation to form intermediate organic compounds that are ultimately mineralized into small-molecule products [124].

TiO_2_ and ZnO are among the most widely used semiconductor materials as photocatalysts [129]. TiO_2_ offers several benefits, including strong reactivity, good photostability, non-toxicity, and moderate cost [130]. ZnO, due to its ability to absorb visible light, significant electron mobility, and low toxicity to humans and marine life, holds a distinct advantage in the commercial degradation of microplastics [131]. For instance, Ebrahimbabaie et al. established a visible-light-driven continuous-flow system using ZnO nanorods immobilized on glass fibers for PP microplastic degradation. Under 0.6 SUN visible-light irradiation (λ > 420 nm) at room temperature (25℃), 65% volume reduction and a carbonyl index of ~ 40 was achieved for PP microplastics (154.8 μm) after 456 h of reaction. The by-products detected in the aqueous phase after photodegradation can be considered to exert relatively low toxic effects on human health and aquatic environments [131]. Nevertheless, this type of photocatalysts face two major core limitations under room temperature experimental conditions for microplastic degradation: limited UV portion of the solar spectrum [132], and a high recombination rate of photogenerated electron-hole pairs [133, 134].

To overcome these limitations of classical semiconductors, researchers have developed a few modification strategies, including heterostructure construction [135], metal/non-metal doping, and surface modification [136], aiming to expand the spectral response range, promote charge separation, and enhance catalytic activity. Currently, most photocatalytic systems are still at the stage of significant oxidation and partial degradation.

Fenton and its derivative technologies are among the most powerful and rapid advanced oxidation processes, with the core reaction involving the use of the Fe^2+^/Fe^3+^-H_2_O_2_ system to catalyze the generation of ·OH [137], which acts on the particle surface, leading to physical and morphological changes such as the formation of wrinkles, cavities, and pores, while also inducing chemical changes through the formation of oxygen-containing functional groups, thereby enhancing its hydrophilicity and surface acidity. The ultimate oxidation products can be CO_2_ [138]. Owing to its strong oxidative capacity, this approach generally exhibits broad applicability to different types of microplastics across diverse environmental matrices, including aquatic systems, soils, and sediments.

However, traditional Fenton-like reactions also have certain limitations, including a narrow pH applicability range, consumption and loss of H_2_O_2_ and iron ions, as well as the potential secondary pollution caused by iron sludge [22, 135]. To overcome these drawbacks, various systems have been derived from the Fenton reaction. For instance, di Luca et al. employed a UV-driven photo-Fenton process for the degradation of PS nanoplastics. The system was operated at pH 3 with iron-catalyzed generation of ·OH under UV irradiation. Within 60 min, the photo-Fenton reaction achieved 90% mineralization of nanoplastics (< 50 nm), accompanied by significant size reduction and fragmentation of the nanoplastics. The hydroxyl radicals initiated oxidative chain scission of the polymer backbone, gradually decomposing PS nanoplastics into small molecular intermediates and finally mineralizing into CO_2_ and H_2_O [139]. In addition, multi-field coupled nano-catalytic pathways have also attracted attention, such as the piezoelectric-photocatalysis-Fenton synergistic system. An internal electric field within the porous structure promotes the separation of photogenerated electrons and holes by driving them in opposite directions, thereby reducing their recombination. This synergistic effect enables the degradation rate of pollutants to far exceed the sum of individual photocatalytic or piezoelectric catalytic processes [140, 141]. For instance, Lu et al. constructed a ternary piezoelectric photocatalyst with a hetero-interpenetrating structure (denoted as Mo_5_Fe_5_). The catalyst in-situ generated H_2_O_2_ via the piezo-photocatalytic synergistic effect and initiated a Fenton-like reaction, achieving a PS degradation efficiency of 58.46% within 30 h [142].

Enzyme-based degradation of M/NPs generally relies on enzyme adsorption onto the plastic surface, whereas nanozyme-mediated degradation is often driven by ROS, making direct adsorption less critical. Moreover, M/NPs produced by different manufacturing processes or subjected to varying environmental and weathering conditions can exhibit distinct surface properties that significantly influence enzyme adsorption and degradation efficiency [33]. Therefore, careful characterization of plastic surface properties is essential for mechanistic understanding and rational optimization of degradation processes. Such characterization can be achieved using surface-sensitive spectroscopic techniques, including X-ray photoelectron spectroscopy (XPS) and infrared (IR) spectroscopy [33, 143].

Although the degradation strategies discussed above have all demonstrated potential for M/NPs treatment, they differ substantially in applicable polymer types, catalytic materials, reaction environments, and degradation outcomes. Table 5 summarizes representative degradation systems reported to date, including enzymes and engineered enzymes, nanomaterial-coupled enzymatic systems, nanozymes, single-atom nanozymes, and advanced oxidation processes. This comparison indicates that M/NPs degradation is governed primarily by polymer chemistry and interfacial accessibility, rather than by catalytic activity alone. For PET, enzymatic systems represent the most chemically selective route because they can depolymerize ester-containing polymers into recoverable monomers. However, their performance is strongly influenced by crystallinity, particle size, pretreatment, reaction medium, enzyme stability, and mass transfer at the enzyme–plastic interface. In contrast, nanozymes, and advanced oxidation-based strategies can broaden degradation toward more chemically inert polymers, but their reported high mass-loss values often rely on oxidative conditions, elevated temperatures, external irradiation, or prolonged reaction times. Importantly, mass loss does not necessarily indicate complete depolymerization or mineralization. Future evaluations should therefore integrate molecular-weight evolution, product distribution, catalyst stability, leaching behavior, product ecotoxicity, and the potential recovery or reutilization of degradation products. Such criteria are necessary to distinguish true polymer degradation from surface oxidation or physical fragmentation and to guide the development of practically relevant M/NPs remediation strategies.

**Table 5:** The different degradation strategies and conditions for microplastics

Degradation strategies	Catalytic materials	Polymer type	Reaction time	Loss weight	References
Enzymes and engineered enzymes	LCC-A2	PET	5.8 h	> 90%	[144]
	FAST-PETase	PET	96 h	40%	[145]
	LCC-ICCG	PET	144 h	76%	[146]
	C09	PET	144 h	100%	[146]
	Cut19	PET	72 h	90%	[147]
	Fe_3_O_4_-PETase	PET	42 h	28.3%	[105]
Nanozymes and SANs	LFMP	PA6	8 h	91.5%	[148]
	LFMP	HDPE	12 h	66.7%	[148]
	LFMP	PP	12 h	78.0%	[148]
	Fe_3_O_4_	HDPE	–	> 90%	[22]
	Fe_3_O_4_	PP	–	> 90%	[22]
	Fe-N_4_	UHMWPE	12 h	> 80%	[118]
Photocatalysis	TiO_2_	HDPE	360 h	78.0%	[22]
	GO/TiO_2_	PE	8 h	50%	[22]

## Challenges, prospects and conclusions

### Challenges and prospects

M/NPs have emerged as pervasive environmental pollutants, making their reliable identification, efficient enrichment, and effective degradation essential for mitigating plastic contamination. Despite current advances, significant challenges remain in translating these approaches into practical environmental applications.

1. **Overcoming complex environmental matrices.** Environmental samples contain high salinity, natural organic matter, colloids, and biomacromolecules that can compromise selectivity, enrichment, and stability [149]. Surface aging and eco-corona formation further alter plastic interfaces, weakening specific binding and increasing nonspecific adsorption. For example, lipid interference in Py-GC-MS analysis of M/NPs in blood can lead to false positives [150]. Therefore, detection strategies should target environmentally aged plastics rather than pristine surfaces and tolerate biofilm or organic coatings. This can be achieved through probes that penetrate or adapt to surface layers, as well as signal amplification, secondary recognition, or multiprobe systems to improve specificity and robustness. Interestingly, recent studies have taken an inverse approach by exploiting the biomolecular composition of microplastic ecological coronas as traceable environmental fingerprints, enabling short period source tracking with reported accuracies of 69–92% [151].

ML further enhances detection in complex matrices by combining preprocessing (e.g., baseline correction and feature extraction) with classification models (e.g., random forests), enabling reliable identification of weak Raman signals [152, 153]. This approach also informs recognition-based microplastic detection: integrating ML with signals from recognition elements (e.g., electrochemical or fluorescence) can improve accuracy and guide the design of higher-performance sensing systems.

In nanomaterial-based enrichment and degradation of microplastics, complex organic matter and colloids in water can induce nanoparticle aggregation, reducing effective interactions and overall efficiency [154]. Common mitigation strategies, such as surface functionalization to adjust charge or steric coatings to prevent aggregation [155], often increase cost and limit scalability. An alternative is to leverage natural organic matter: for example, humic substances can stabilize nanoparticles and enhance dispersion under certain conditions [156]. Designing humic-like coatings or controlling their adsorption may therefore offer a cost-effective approach to mitigate aggregation.

A more practical route to overcome matrix interference is to shift from single-probe recognition to matrix-adaptive, orthogonally validated detection workflows. For example, porous plasmonic substrates with hydrophobic trapping layers can simultaneously enrich microplastics and generate SERS fingerprints, while self-attention-based neural networks can deconvolute weak and overlapping spectra from multi-polymer mixtures in the presence of environmental interferents [82, 83]. Such designs suggest that anti-interference capability should be evaluated not only by signal intensity, but also by polymer-level classification accuracy, recovery in matrix-matched samples, false-positive rates, and robustness toward algae, proteins, lipids, humic substances, salts, and mineral particles.

For enrichment, future efforts should emphasize recoverable, immobilized, or flow-through platforms rather than freely dispersed nanomaterials in batch tests [157]. Magnetic adsorbents, bio-based filters, fixed photocatalyst films, and continuous-flow reactors offer practical routes to improve material recovery, reduce secondary release, and enable long-term operation in real water matrices. Their performance should be assessed by treatment volume, residence time, capture efficiency, material dosage, regeneration stability, and activity decay. For degradation, mass loss or particle fragmentation alone is insufficient; molecular-weight reduction, TOC removal, CO₂ formation, additive release, catalyst leaching, and product ecotoxicity should be monitored to verify true detoxification or mineralization [135].

Due to the differences between nanoplastics and microplastics in terms of colloidal stability, mobility, surface area-to-volume ratio, and eco-corona formation, their identification, enrichment, and degradation strategies exhibit pronounced size-dependent variations [158]. Identification of nanoplastics typically relies more on labeling-enhanced techniques, high-sensitivity separation-coupled detection, and single-particle analytical approaches [159]. Their enhanced suspension stability, colloidal behavior, and surface reactivity necessitate enrichment strategies based on interfacial functionalized adsorption, membrane separation, or electro-driven capture for targeted isolation [160]. In terms of degradation, the increased specific surface area at the nanoscale may enhance surface activation; however, the rapid formation of eco-corona, re-aggregation, and interactions with natural organic matter can mask reactive sites and alter reaction pathways, resulting in degradation behaviors that differ from those of microplastics in both kinetics and regulatory mechanisms [161]. Therefore, nanoplastics should be considered as a distinct size regime when developing identification, enrichment, and degradation strategies.

(2) **Difficulties in implementation.** Although recent studies increasingly incorporate real environmental matrices, translating these systems from laboratory conditions to field deployment remains challenging. Key barriers include economic feasibility at scale and potential ecological risks associated with nanomaterials used for microplastic detection, degradation, and quantification.

Biomolecular recognition elements, such as peptides and aptamers, often involve relatively high costs, which can limit their practicality in environmental applications where cost-efficiency is critical. Similarly, widely used nanomaterials (e.g., carbon nanotubes, graphene oxide, and noble metal nanoparticles) are expensive to produce, further constraining large-scale deployment. Thus, material selection must balance cost with performance.

Environmental risks also arise throughout the lifecycle of nanomaterial-based systems, including synthesis, release [162], transformation [162], ecotoxicity [163], interactions with co-pollutants [164], and secondary pollution [15]. These risks can be mitigated through safer design, material immobilization or recovery, greener synthesis, and life cycle assessment integrated with regulatory frameworks [165]. Bio-based flow-through filters, substrate-bound photocatalyst films, magnetic composites, and electrode-supported catalytic systems provide practical routes to reduce material loss, facilitate separation, and improve compatibility with existing water-treatment units. In this context, scalability should not be judged only by removal or degradation efficiency, but also by material dosage, treated volume, residence time, regeneration stability, energy input, and performance decay in real water matrices [131].

Environmental safety should likewise be evaluated from a life cycle assessment. Nanomaterial enabled platforms may introduce risks through particle release, metal leaching, transformation products, spent adsorbents, catalytic residues, or degradation by-products [166]. Therefore, safe design principles should be combined with immobilization, green synthesis, and end-of-life management. Integrating life cycle assessment and techno-economic analysis with standardized risk assessment will be essential for identifying platforms that are not only effective, but also scalable, recyclable, and environmentally acceptable. Future efforts should therefore prioritize not only performance but also cost-effectiveness, environmental compatibility, recyclability, and standardized risk evaluation.

(3) **Functional coupling of recognition, enrichment and degradation.** Microplastic detection, separation, and degradation technologies are still developed largely in isolation, limiting their translation into integrated, scalable solutions. A promising direction is to couple molecular recognition with selective enrichment. While biomolecular recognition elements are widely used in sensing, their potential for guiding selective capture remains underexplored. Integrating these elements with enrichment materials (e.g., magnetic nanoparticles or porous substrates) could enable recognition-mediated capture directly from complex matrices [43]. For instance, peptide-functionalized magnetic nanoparticles have enabled rapid microplastic detection, and multifunctional magnetic adsorbents combining capture, catalytic activity, and machine learning allow simultaneous enrichment, signal generation, and accurate identification [167]. Equally important is coupling enrichment with catalytic transformation. Hybrid materials can integrate adsorption with photocatalytic or Fenton-like degradation, enabling capture and breakdown within a single system [168]. Enrichment enhances local concentration and mass transfer, promoting degradation, though challenges such as catalyst deactivation, fouling, and incomplete mineralization remain.
(4) **Nanoplastics should be treated as a distinct interfacial regime rather than a simple extension of microplastics.** Many laboratory nanoplastic models are prepared by bottom-up methods, such as emulsion polymerization or nanoprecipitation, yielding monodisperse 50–500 nm spheres with surfactant residues or ionizable groups such as –COOH and –NH_2_ [33]. These engineered surfaces are often highly charged and colloidally stable, whereas environmentally generated nanoplastics arise mainly from top-down fragmentation of aged polymers and exhibit irregular morphology, partial oxidation, surfactant-free interfaces, and matrix-conditioned surface coatings [158, 169].

Natural organic matter, inorganic colloids, ions, and biomolecules can further alter their ζ-potential, hydrodynamic size, aggregation state, and eco-corona, thereby masking the polymer interface targeted by antibodies, aptamers, peptides, dyes, or nanomaterial probes [170]. Therefore, recognition strategies validated only with pristine model nanospheres should be interpreted cautiously and further tested using aged, fragmented, and environmentally conditioned nanoplastics.

These size- and surface-dependent effects also influence nanoplastic enrichment. In magnetic capture, surfactant-stabilized nanoplastics may resist heteroaggregation with Fe_3_O_4_ under low ionic strength because of electrostatic repulsion, whereas organic matters and divalent cations can promote cation bridging in natural waters. For MOF-based capture, most nanoplastics are much larger than the micropores of typical frameworks such as ZIF-8 or UiO-66, restricting adsorption mainly to external surfaces rather than internal porosity. Hierarchical mesoporosity, defect engineering, and tailored surface chemistry are therefore needed to match nanoplastic dimensions and interfacial properties [65].

Nanoplastic degradation is likewise dominated by surface-controlled reactions. For hydrolysable polymers such as PET, the nanoscale size increases the accessible polymer–water–enzyme interface and can accelerate hydrolysis, as shown by the rapid degradation of 50–100 nm PET nanoplastics by engineered PET hydrolases [171]. However, smaller size alone does not ensure efficient depolymerization, because enzyme adsorption, particle aggregation, surface coverage, and enzyme distribution strongly regulate the apparent reaction rate. Thus, particle-size reduction should not be used as the sole evidence of degradation; released monomers or oligomers, bond-cleavage signals, molecular-weight changes, and mineralization indicators should also be monitored [172]. In enzyme–nanomaterial hybrids, pore or network dimensions must be matched to nanoplastic size to avoid burying enzymes in inaccessible internal sites. Interconnected porous hydrogels and magnetic enzyme carriers provide promising routes by improving enzyme stability, substrate access, recovery, and reuse [173]. Overall, nanoplastic identification, enrichment, and degradation require strategies specifically designed for their colloidal behavior, dynamic surface chemistry, and eco-corona formation.

### Conclusions

This review highlights that advances in M/NPs research remain fragmented across recognition, enrichment, and degradation, despite their shared reliance on interfacial processes. Rather than further developing isolated technologies, a key priority is to understand how interfacial interactions can be deliberately coupled across these steps. Such integration needs to be evaluated under environmentally relevant conditions, where aging, eco-corona formation, and matrix interference can significantly alter interfacial behavior and reduce performance. At the same time, practical implementation is constrained by cost, material stability, and potential environmental risks of nanomaterials, factors often overlooked. Progress will depend less on introducing new material classes and more on establishing quantitative relationships between interfacial structure, binding, and catalytic outcomes. This approach may provide a more practical and scalable path for monitoring and managing M/NPs.

## Data Availability

No primary research results, software or code have been included and no new data were generated or analyzed as part of this review.
